# Genome-wide association studies of grain yield and quality traits under optimum and low-nitrogen stress in tropical maize (*Zea mays* L.)

**DOI:** 10.1007/s00122-022-04224-7

**Published:** 2022-09-21

**Authors:** Noel Ndlovu, Charles Spillane, Peter C. McKeown, Jill E. Cairns, Biswanath Das, Manje Gowda

**Affiliations:** 1 Plant & AgriBiosciences Research Centre, Ryan Institute, National University of Ireland Galway, University Road, Galway, H91 REW4 Ireland; 2 International Maize and Wheat Improvement Center (CIMMYT), P.O. Box 1041-00621, Nairobi, Kenya; 3 International Maize and Wheat Improvement Center (CIMMYT), P.O. Box MP163, Harare, Zimbabwe

## Abstract

**Key message:**

**Genome-wide association study (GWAS) demonstrated that multiple genomic regions influence grain quality traits under nitrogen-starved soils. Using genomic prediction, genetic gains can be improved through selection for grain quality traits.**

**Abstract:**

Soils in sub-Saharan Africa are nitrogen deficient due to low fertilizer use and inadequate soil fertility management practices. This has resulted in a significant yield gap for the major staple crop maize, which is undermining nutritional security and livelihood sustainability across the region. Dissecting the genetic basis of grain protein, starch and oil content under nitrogen-starved soils can increase our understanding of the governing genetic systems and improve the efficacy of future breeding schemes. An association mapping panel of 410 inbred lines and four bi-parental populations were evaluated in field trials in Kenya and South Africa under optimum and low nitrogen conditions and genotyped with 259,798 SNP markers. Genetic correlations demonstrated that these populations may be utilized to select higher performing lines under low nitrogen stress. Furthermore, genotypic, environmental and GxE variations in nitrogen-starved soils were found to be significant for oil content. Broad sense heritabilities ranged from moderate (0.18) to high (0.86). Under low nitrogen stress, GWAS identified 42 SNPs linked to grain quality traits. These significant SNPs were associated with 51 putative candidate genes. Linkage mapping identified multiple QTLs for the grain quality traits. Under low nitrogen conditions, average prediction accuracies across the studied genotypes were higher for oil content (0.78) and lower for grain yield (0.08). Our findings indicate that grain quality traits are polygenic and that using genomic selection in maize breeding can improve genetic gain. Furthermore, the identified genomic regions and SNP markers can be utilized for selection to improve maize grain quality traits.

**Supplementary Information:**

The online version contains supplementary material available at 10.1007/s00122-022-04224-7.

## Introduction

Maize (*Zea mays* L.) yields in sub-Saharan Africa (SSA) are amongst the lowest in the world (FAO 2021). Average yields in this region range from 1 to 3 t ha^−1^, well below the global average of around 5 t ha^−1^ (Prasanna et al. 2020, 2021). Over a quarter of households in SSA are deemed persistently food insecure, with that figure climbing to 40% during the dry season (Fraval et al. 2019). Furthermore, demand for maize in this region is expected to triple by the year 2050 as a result of rapid population growth (Ekpa et al. 2018). While drought stress and increased climate variability are linked to low yields, low fertilizer use is also a key driver of the maize yield gap in this region (Tittonell and Giller 2013). While average N fertilizer use in SSA by smallholder farmers has increased over the past decade, however it remains very low at 17.9 kg N ha^−1^ (Jayne and Sanchez 2021). This difference is particularly pronounced on female managed plots which tend to receive less nitrogen inputs than male management plots within a farm (Cairns et al. 2021; Farnworth et al. 2017).

For vulnerable populations, increased dietary diversity and consumption of nutrient-rich food is essential for increased nutrition (Poole et al. 2021). However, in more than 20 countries in SSA, maize accounts for more than 30% of calories consumed (Goredema-Matongera et al. 2021). In Lesotho, Malawi, Zambia, and Zimbabwe, the average per capita consumption of maize is more than 100 kg per person per year (i.e., 300 g per day), roughly half of daily calorie intake (Cairns et al. 2021; Prasanna et al. 2021). High-quality protein sources include eggs, meat, and dairy products, but vulnerable populations in SSA have limited access to these foods and rely heavily on maize as a primary source of protein (Nuss and Tanumihardjo 2011). Average protein supply from maize is 10 g per capita per day in SSA, with up to 35 g per capita per day in southern Africa (FAO 2021). In Burkina Faso Eswatini, Ethiopia, Lesotho, Malawi, Mozambique, Nigeria, Tanzania, Togo, Zambia and Zimbabwe, maize provides a greater source of protein than protein derived from animal sources (FAO 2021).

Generally, maize grain has low oil (4.4%) and protein (9.1%) contents but a relatively high starch content (73.4%) basing on dry matter measurements (Dei 2017). In temperate maize, breeding has led to significant increase in grain yield. However, grain protein content is estimated to have decreased 0.3% per decade and grain starch content has increased at 0.3% per decade (Duvick 2005). Grain oil content has also reduced over time in temperate maize (Scott et al. 2006). Grain quality is linked closely linked to soil quality (Wood et al. 2018). Maize protein content increases as the level of N applied increases (Zhang et al. 2020). To date, studies looking at the effect of reduced N level on grain quality have used significantly higher levels of N than is relevant to smallholder farmers in SSA. Under low nitrogen stress in SSA environments, previous maize grain quality assessment studies, such as Abu et al. (2021), Ngaboyisonga and Njoroge (2014), and (Oikeh et al. 1998), used N rates ranging from 30 to 120 kg ha^−1^. Such application rates are higher than the average N application rates (which is between 12 and 16 kg ha^−1^ (Heffer and Prud’homme 2015; Sheahan et al. 2014) in smallholder agriculture in SSA. Incorporating grain quality traits into breeding can involve significant costs associated with grain nutrient analyses, morphological characterizations, and associated trait complexities stymie progress in improving maize grain quality. The high-cost requirement of measuring grain quality traits stems from the wet chemistry procedures required to create near-infrared spectroscopy (NIRS) calibration curves, which are relatively expensive. There is a significant opportunity to assess and employ the potential of genomic selection in the improvement of grain quality traits in maize. This reinforces the need for more rapid progress to improve maize grain quality components (particularly, protein, starch, and oil content) through accelerated and more efficient breeding enabled by molecular markers.

The integration of genomic tools such as genomic-wide association studies (GWAS), linkage mapping and genomic selection (GS) with traditional breeding approaches have increased the efficiency of grain quality and Low N tolerance selection. These techniques have been used to identify causal variants for Low N tolerance. Linkage mapping is a common method for locating quantitative trait loci (QTL) based on a segregating population derived from a cross of two parental lines with significantly divergent phenotypes (Xiao et al. 2017). Linkage mapping can be combined with GWAS to provide a more comprehensive strategy for identifying markers linked with a trait of interest, by the discovery of trait linked markers by GWAS and their validation through linkage mapping. The most robust markers can then be employed for marker-assisted selection (MAS). However, the accuracy with which genetic maps are constructed is dependent on the mapping population since doubled haploid (DH) lines, near-isogenic lines (NILs), recombinant inbred lines (RILs), and backcross lines are extremely effective yet to date labour-intensive and time-consuming to generate (Rao et al. 2021). GS has been highlighted as a powerful option for selecting polygenic traits that are difficult to select through MAS. GS can accomplish this by employing genome-wide dense markers for predictions, and therefore can support association analyses to determine the genetic basis of grain yield and quality related traits (Bentley et al. 2014). Galli et al. (2020) reported that GWAS could identify both additive and dominant genetic effects that influence Low N tolerance in maize.

To understand how low N stress affects grain yield and grain quality traits such as grain protein, starch, and oil content, this study was performed using a tropical maize population under low N and optimum conditions across multi-location field trials in Kenya and South Africa. The study had the objectives of (i) Assessing the genetic architecture of low soil-N tolerant maize test crosses using their responses to grain quality and yield traits under two management conditions (optimum and low soil N); (ii) Identifying the significant quantitative trait nucleotides (QTNs) and putative candidate genes and QTLs for quality traits in tropical maize germplasm tested in multiple locations; and (iii) Assessing the potential of utilizing GS in the improvement of grain quality traits.

## Materials and methods

### Germplasm, experiment design and management

An association mapping panel of 410 tropical maize lines developed under CIMMYT’s Improved Maize for African Soils (IMAS) project (Ertiro et al. 2020a) in collaboration with the Kenya Agricultural and Livestock Research Organisation (KALRO) and Agricultural Research Council (ARC, South Africa) was used. The study also evaluated two DH populations from CIMMYT’s Heterotic group B (221 lines of CML550/CML504, 115 lines of CML550/CML511), and two DH population from CIMMYT’s Heterotic group A (175 lines of CML505/LaPostaSeqC7-F64-2-6-2-2 and 131 lines of CML536/LaPostaSeqC7-F64-2-6-2-2) (Ertiro et al. 2020b). Test cross hybrids were generated by crossing all inbred lines with a broadly adapted CIMMYT maize inbred line tester from the opposite heterotic group.

All testcross progenies for both association panel and DH populations were evaluated in three optimal and six low N-stressed sites (Table 1). Experiments conducted in the same site over several years were classified as separate environments (Das et al. 2019; Ertiro et al. 2020a). Kiboko lies within longitudes 37.7235°E and latitudes 2.2172°S, at an elevation of 975 m above sea level. The station receives between 545 and 629 mm of rainfall split in two seasons and lies in a hot, semi-arid region with annual temperature ranging from 16.0 to 33.6 °C. The soils are well drained, very deep, dark reddish brown to dark red, friable sandy clay to clay (Acri-Rhodic Ferrassols) developed from undifferentiated basement system rocks, predominantly banded gneisses (Ertiro et al. 2022). Other location Embu lies at an elevation of 1350 m above sea level and receives an average of 893 mm of rainfall and lies in the foothills of Mount Kenya with annual temperature ranging from 15.0 to 27.9 °C. Cedara research station in South Africa lies 1037 m above sea level and receives an average of 990 mm of rainfall annually. All testcross progenies were evaluated in an alpha-lattice design with two replications. The sites for low N trials were depleted for soil N content by growing sorghum for several years without applying any external N fertilizer. Experiments were planted in one-row plots, with a planting density of 5.33 plants/m^2^ (Kenya and South Africa). In each location, two seeds per hill were sown, then thinned to one after emergence. At planting, triple phosphate (46% P_2_O_5_) was applied to the low N trials at the rate of 50 kg P_2_O_5_/ha. On optimum trials, diammonium phosphate (DAP) fertilizer was used at the rate of 54 kg N per hectare. Optimum trials were top-dressed with urea fertilizer at the rate of 138 kg N per hectare three weeks after planting. All trials under both optimum and low-N were irrigated as required to avoid any moisture stress. Trials under both conditions were kept weed-free and other standard agronomic practices were conducted.

**Table 1:** Description of the field trials used in the study

Country	Site name	Management options	Germplasm used	Season
Kenya	Embu	Low N	IMAS	2011B, 2012A
		Low N	CML550/CML504	2014A
		Low N	CML550/CML511	2014A
	Kiboko	Optimum	IMAS	2011A, 2012A
		Low N	IMAS	2011A, 2011B, 2012A
		Optimum	CML505/LaPostaSeqC7-F64-2-6-2-2	2015B
		Low N	CML505/LaPostaSeqC7-F64-2-6-2-2	2015A, 2015B
		Optimum	CML550/CML511	2014A
		Low N	CML550/CML504	2015A, 2015B
		Low N	CML550/CML511	2015A
		Low N	CML536/LaPostaSeqC7-F64-2-6-2-2	2014A, 2015A, 2015B
South Africa	Cedara	Optimum	IMAS	2011B
		Low N	IMAS	2011B

*A   main season;* B * off-season

### Measurements of grain yield and quality traits

Data were recorded for grain yield and quality traits (i.e., protein, oil, and starch contents). Shelled grain yield was measured in kilograms (kg) and converted to tons per hectare and reported at 12.5% moisture. Protein, starch, and oil content were measured using a FOSS Infratec TM 1241 from 500-g samples of grain taken from each plot and are reported as a percentage of whole grain. The FOSS Infratec is a non-destructive whole-grain analyzer that uses near-infrared reflectance (NIR) to estimate quality parameters. Five 100-g subsamples were assayed and the mean reading for each parameter was reported per plot.

### Phenotypic data analysis

Analyses of variance for each biparental population and IMAS panel at each and across locations under optimum and Low-N conditions was performed in the R program embedded in META-R (Alvarado et al. 2020) and ASREML-R (Gilmour et al. 2009). The linear mixed model with the restricted maximum likelihood (REML) was used to calculate all variance components. The study treated replication as fixed effect and all other treatment effects as random. On an entry-mean basis, the broad-sense heritability (*H*^*2*^) was estimated using the genotypic to phenotypic variance ratio from the derived variance components. Furthermore, to determine the genotypic effects of the investigated lines for each and across environments, best linear unbiased estimation (BLUE) and best linear unbiased prediction (BLUP) were obtained. For GWAS and linkage mapping, BLUPs were used. On the other hand, BLUEs were used for GS analyses. The classification of the genotypic correlation coefficients followed the guidelines provided by Profillidis and Botzoris (2019). To determine the impact of low N stress on the aforementioned traits, we used a *t*-test to compare the mean values of the two management conditions. We also determined the percentage change (i.e., decrease or increase) in grain quality trait performance.

### Genotyping-by-sequencing (GBS)

DNA was extracted according to the CIMMYT high-throughput mini-prep Cetyl Trimethyl Ammonium Bromide (CTAB) method (Semagn (2014). Following the protocol presented in Elshire et al. (2011), maize DNA samples were genotyped using a restriction enzyme (*Ape*KI) and 96-plex multiplexing at the Institute of Biotechnology at Cornell University, USA. The Institute of Genomic Diversity (IGD) at Cornell provided raw GBS data for a maximum of 955,120 SNP loci spread throughout the 10 maize chromosomes (Ertiro et al. 2020a). Raw data was filtered for linkage mapping according to the criteria used in Ertiro et al. (2020a) of > 10 percent minor allele frequency (MAF) and no missing data. Furthermore, the genotype data were filtered for GWAS using the Trait Analysis by Association, Evolution, and Linkage (TASSEL v.5.2.7.2, Bradbury et al., 2007) software, with a baseline count of SNPs on 90% and a MAF of > 5% of the sample size as presented in Ertiro et al. (2020a). Principal Component Analysis (PCA) was carried out in TASSEL (v.5.2.7.3), as were genetic distances and kinship.

### Genome-wide association study analysis

In natural populations or association panels, the population structure and relative kinship cause high level of spurious positives during association studies. To assess the effect of population structure, PCA, and the relative kinship (K) on association results in IMAS panel, we used the following statistical models: (1) uncorrected genotypic data only (GLM with G only); (2) GLM with PCA + G; (3) a mixed linear model (MLM) with PCA + K + G; (4) and FarmCPU model. G = genotype (fixed), PCA = three principal components (fixed), K = kinship matrix (random). The R package ‘FarmCPU-Fixed and random model Circulating Probability Unification’ (Liu et al. 2016) was used for GWAS analysis for all traits. FarmCPU utilized the first three PCs derived by TASSEL as input for GWAS. The kinship was computed using FarmCPU’s default kinship algorithm as presented in Ertiro et al. (2020a). The analysis was performed with maxLoop of five, *p* threshold of 0.1, QTN threshold of 0.1 and MAF threshold of 0.05. The maxLoop refers to the total number of iterations used. The *p* threshold, QTN threshold and MAF threshold refers to *p* values selected into the model for the first iteration, the *p* value selected into the model from the second iteration and the minimum MAF of SNPs used in the analysis. False discovery rate threshold of 0.1 was used to set a significant level in Manhattan plots. The Manhattan and quantile–quantile (QQ) plots, GWAS findings, and a table of marker effects of user-provided variables were all produced by the FarmCPU using the “GAPIT” function. To annotate putative candidate genes for traits under study, the physical positions of the significant SNPs were compared with the Maize B73 reference genome version 2 (RefGen_v2), available at the MaizeGDB database (www.maizegdb.org) and functional gene annotations were retrieved from http://ensembl.gramene.org/Zea_mays. The presence of the protein-coding genes was searched within the range of 20 kb (10 kb upstream and downstream) in the vicinity of the detected significant SNPs.

### QTL mapping and genomic prediction

The four DH populations were genotyped with GBS and data was further filtered to a manageable size using TASSEL software with > 0.10 MAF, < 5% heterozygosity, and 90% the minimum count of the total size (Bradbury et al. 2007; Sitonik et al. 2019). In all the populations, homozygous marker loci for both parents and uniformly distributed polymorphic markers between parents were retained. Linkage maps were constructed by using QTL IciMapping version 4.1 (Meng et al. 2015) in all four DH populations. BIN is an inbuilt tool implemented in QTL IciMapping was used to remove the highly correlated SNPs. This resulted into retain 2699, 1962, 1985 and 2086 high-quality SNPs in CML550/CML504, CML550/CML511, CML505/LaPostaSeqC7-F64-2-6-2-2 and CML536/LaPostaSeqC7-F64-2-6-2-2, respectively. These SNPs were used to construct linkage maps using the MAP function. IciMapping used the grouping, ordering, and rippling steps to construct a linkage map. The ICIM is an effective two-step statistical approach that allows separation of co-factor selection from interval mapping process, in order to control the background effects and improve mapping of QTL with additive and dominance effects. The Kosambi genetic distance mapping function which assumes that recombination events influence the occurrence of adjacent recombination was used.

For QTL mapping, we used IciMapping 4.1 based on biparental populations (BIPs) module with inclusive composite interval mapping (Meng et al. 2015). BLUP values across environments for each trait in each the DH populations were used in QTL detection analysis. The mapping populations were grouped by the SNPs and the significant difference between the means (*P*-value < 0.0001) was detected based on the markers that were linked to a QTL controlling the selected target trait (Collard et al. 2005). The highest peak of one LOD that supports the confidence interval was used to declare the significance of the QTL map position on both sides of the QTL (Hackett 2002). A LOD threshold of 3.0 with a scanning step of 1 cM were used to declare significant QTL. Stepwise regression was adopted to determine the percentages of phenotypic variance explained (*R*^2^) by individual QTL and additive effects at LOD peaks. The phenotypic variation explained (PVE) by each QTL and together for all QTLs for each trait was estimated. The origin of the favourable allele for each trait was identified based on the sign of the additive effects of each QTL.

BLUEs across environments for each trait in each population were used in the GS analysis. The Ridge-regression BLUP (RR-BLUP, Zhao et al. 2012) with fivefold cross-validation for each trait was used for the analysis. A sample of 4000 SNPs with all data values, equally distributed throughout the genome, and MAF > 0.05 was chosen from the GBS data for the IMAS panel and all four DH populations. Individual DH population and the IMAS set were sampled to form a training and prediction set. The prediction accuracy was calculated as the correlation between the observed phenotypes and genomic estimated breeding values (GEBVs) divided by the square root of heritability (Dekkers 2007). In each population, 100 iterations were done for the sampling of the training and validation sets.

## Results

### Effect of low N stress on grain yield and quality traits

**Fig. 1:**
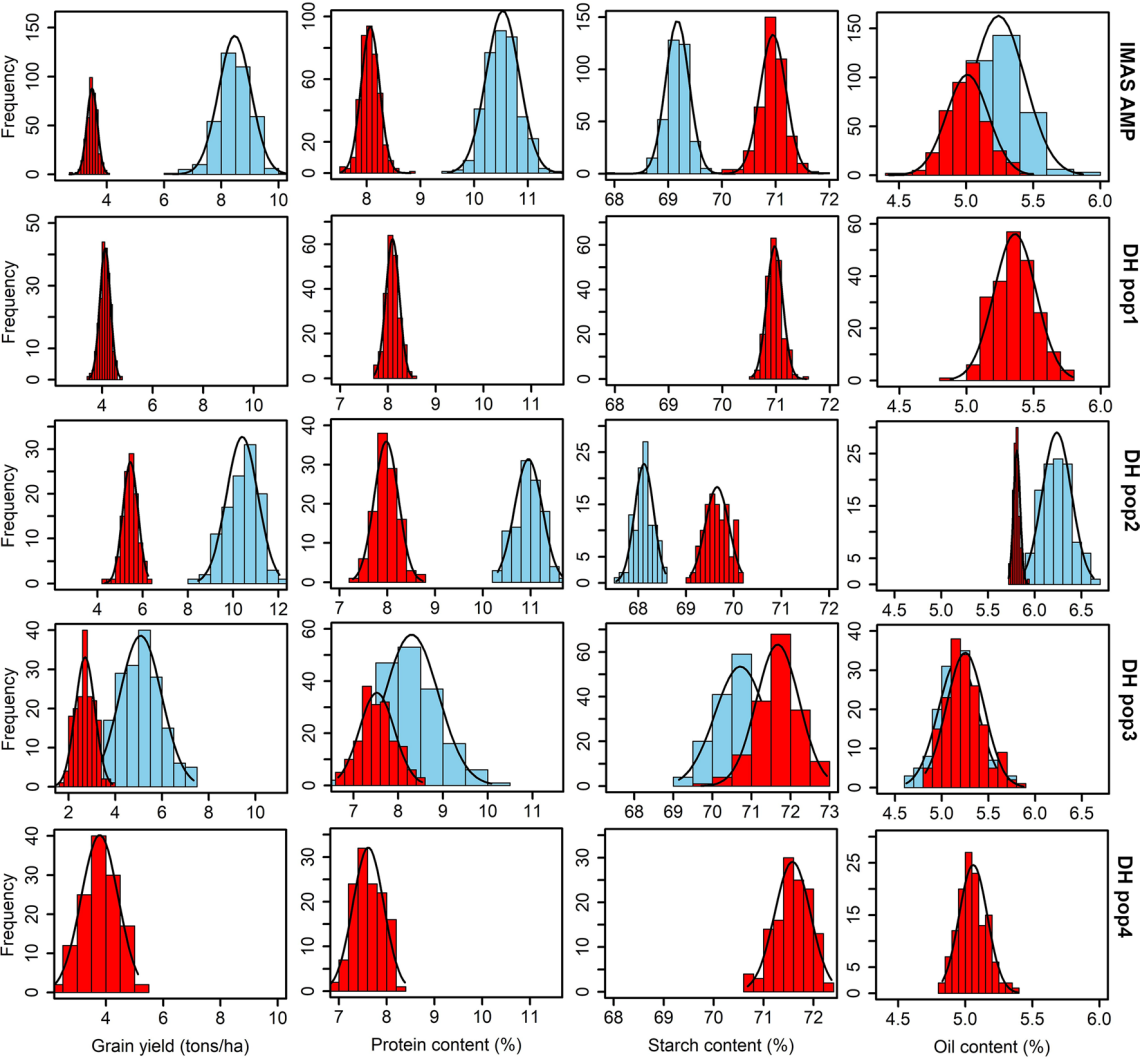
Phenotypic distribution for grain yield and quality traits evaluated under optimum and Low N stress conditions. The sky blue and red colour plots represent the trials conducted under optimum and low N stress conditions, respectively. DH pop1 = CML550/CML504; DH pop2 = CML550/CML511; DH pop3 = CML505/LaPostaSeqC7-F64-2-6-2-2; and DH pop4 = CML536/LaPostaSeqC7-F64-2-6-2-2

There was significant variation in protein, starch and oil content, and grain yield within all four biparental DH populations and the IMAS panel under optimum and low N stress conditions (Fig. 1 and Table 2). In the IMAS panel and CML505/LaPostaSeqC7-F64-2-6-2-2 DH pop, yield under low N stress was reduced by 59% and 48%, respectively. In DH pop CML550/CML511, the mean yield under low N stress was 5.45 t ha^−1^; however, this was a reduction of 47% relative to optimal conditions. Low N stress significantly (*p* < 0.01) reduced protein and oil content (except in DH pop CML505/LaPostaSeqC7-F64-2-6-2-2) but had no significant effect on starch content. Although the level of N stress and therefore the reduction in grain yield was the lowest in DH pop CML550/CML511, both protein and oil content had the largest reduction in this population under low N stress.

**Table 2:** Genetic parameters for IMAS panel and four DH Populations evaluated under optimum and low N stress conditions in multiple environments

	Optimum					Low N				
	Mean	*σ*^2^_G_	*σ*^2^_GE_	*σ*^2^_e_	*h*^*2*^	Mean^a^	*σ*^2^_G_	*σ*^2^_GE_	*σ*^2^_e_	*H*^*2*^
*IMAS Panel*										
Grain yield (t/ha)	8.47	0.59**	0.38**	1.37	0.62	3.49**	0.08**	0.13**	0.67	0.50
Protein content (%)	10.53	0.15**	0.02**	0.33	0.71	8.07**	0.06**	0.08**	0.33	0.59
Starch content (%)	69.17	0.10**	0.02**	0.28	0.55	70.95*	0.11**	0.07**	0.53	0.62
Oil content (%)	5.24	0.05**	0.01**	0.03	0.86	5.01*	0.04**	0.03**	0.06	0.72
*DH pop1 (CML550/CML504)*										
Grain yield (t/ha)	–	–	–	–	–	4.12	0.17**	0.00	0.98	0.41
Protein content (%)	–	–	–	–	–	8.10	0.06**	0.03**	0.19	0.47
Starch content (%)	–	–	–	–	–	70.97	0.08**	0.00	0.51	0.38
Oil content (%)	–	–	–	–	–	5.36	0.03**	0.00	0.03	0.79
*DH pop2 (CML550/CML511)*										
Grain yield (t/ha)	10.40	0.77**	–	2.10	0.42	5.45**	0.27**	0.11**	0.77	0.52
Protein content (%)	10.96	0.12**	–	0.10	0.72	7.98**	0.12**	0.00	0.23	0.67
Starch content (%)	68.12	0.08**	–	0.15	0.52	69.65*	0.12**	0.00	0.30	0.62
Oil content (%)	6.24	0.03**	–	0.02	0.79	5.81*	0.01*	0.04**	0.05	0.20
*DH pop3 (CML505/LaPostaSeqC7-F64-2-6-2-2)*										
Grain yield (t/ha)	5.08	0.07*	–	0.17	0.45	2.70**	0.03*	0.09**	0.30	0.21
Protein content (%)	8.21	0.17**	–	0.21	0.61	7.52**	0.05*	0.07**	0.26	0.33
Starch content (%)	70.96	0.07*	–	0.61	0.18	71.68*	0.09**	0.07**	0.51	0.34
Oil content (%)	5.05	0.02*	–	0.04	0.48	5.25*	0.03*	0.00	0.05	0.66
*DH pop4 (CML536/LaPostaSeqC7-F64-2-6-2-2)*										
Grain yield (t/ha)	–	–	–	–	–	3.78	0.21**	0.12**	0.81	0.54
Protein content (%)	–	–	–	–	–	7.61	0.05*	0.06**	0.17	0.48
Starch content (%)	–	–	–	–	–	71.58	0.02*	0.09**	0.40	0.18
Oil content (%)	–	–	–	–	–	5.06	0.02*	0.01*	0.04	0.66

*, **Significant at *p* < 0.05 and *p* < 0.01 level, respectively; ^a^mean comparison with *t* test, *, **significant at *p* < 0.05 and *p* < 0.01 level, respectively

The genotypic, environmental, and genotype-environment interaction effects (G, E and G x E, respectively) were significant at *p* ≤ 0.05 for yield and quality traits (Table 2). For protein, starch, and oil content under optimal conditions, the magnitude of genotypic variance was greater compared to low N stress conditions. Under low N conditions, the effect of genotype, environment, and G x E interactions on oil content was significant across all genotypes tested. Interestingly, under the same conditions, the genotypic effects on protein content were only significant in DH pops CML505/LaPostaSeqC7-F64-2-6-2-2 and CML536/LaPostaSeqC7-F64-2-6-2-2. The G x E effects on protein content and starch content in DH pop CML550/CML511 were significant. The zero estimates of G x E interactions for starch and protein content (observed on DH pop CML550/CML511 under low N stress) indicate that genotypic performance for these traits was stable across the tested environments. *H*^*2*^ values of each trait under both optimal and low N stress are presented in Table 2. In general, *H*^*2*^ of all traits was lower under low N stress than optimal, with the exception of starch content which increased under low N stress across all populations.

Grain yield was negatively correlated with protein content across populations, regardless of N stress level (Table 3). Similarly, starch content showed a negative correlation with protein and oil content across genotypes and management options. A weak positive correlation was reported between protein and oil content across populations and N levels. The only exceptions were in DH pop CML550/CML504 under optimum conditions where oil content was significantly (at *p* < 0.01) and negatively correlated to protein content (*r* = − 0.92**).

**Table 3:** Genetic correlations between BLUPs of grain yield and grain quality traits evaluated under optimum and low N stress management

	Grain yield	Protein content	Starch content	Oil content
IMAS panel				
Grain yield	–	** − 0.03**	0.28*	**0.01**
Protein content	− 0.41**	–	− 0.30**	0.10*
Starch content	*0.01*	− 0.54**	−	− 0.18**
Oil content	− 0.17*	*0.07*	− 0.65**	–
DH pop1 (CML550/CML504)				
Grain yield	–			
Protein content	− 0.10*	–		
Starch content	− 0.39**	− 0.37**	–	
Oil content	0.21**	*0.03*	− 0.84**	–
DH pop2 (CML550/CML511)				
Grain yield	–	− 0.21*	0.17*	0.18*
Protein content	− 0.24**	–	− 0.78**	**0.09**
Starch content	* − 0.12*	− 0.61**	–	− 0.72**
Oil content	0.25**	− 0.92**	− 0.95**	–
DH pop3 (CML505/LaPostaSeqC7-F64-2-6-2-2)				
Grain yield	–	− 0.43**	0.52**	− 0.36**
Protein content	− 0.24**	–	− 0.53**	0.46**
Starch content	0.30**	− 0.23*	–	− 0.97**
Oil content	*0.02*	*0.00*	− 0.84**	–
DH pop4 (CML536/LaPostaSeqC7-F64-2-6-2-2)				
Grain yield	–			
Protein content	− 0.29**	–		
Starch content	0.26**	− 0.13*	–	
Oil content	− 0.20*	*0.00*	− 0.95**	–

Values in bold cells represent genetic correlations between traits under low N stress, values in italic cells represent genetic correlations between traits under optimum conditions

*, **Significant at *p* < 0.05 and *p* < 0.01 level, respectively

Protein content had a negative correlation with grain yield (*r* = − 0.41**) and starch content (*r* = − 0.54**) in the IMAS panel under optimum conditions. Similarly, a weak positive correlation was observed in the IMAS panel, DH pops CML550/CML504 and CML505/LaPostaSeqC7-F64-2-6-2-2 between protein content and oil content under low N stress. Starch and oil contents were shown to be significantly (*p* < 0.05) and negatively correlated under optimum (*r* = − 0.65) and low N stress (*r* = − 0.18) in the IMAS panel. The genotypic correlation coefficients for DH pops CML505/LaPostaSeqC7-F64-2-6-2-2 and CML536/LaPostaSeqC7-F64-2-6-2-2 showed that oil content under optimum conditions had no correlation with protein content (*r* = 0.00). As demonstrated in Table 3, further significant (*p* < 0.05 and *p* < 0.01) trait correlations were established among the phenotypic parameters measured across the management conditions for each set of genotypes. The observed correlations between grain quality and yield can be useful for selection decisions or trade-offs in genotypic selection.

### GWAS analyses

**Fig. 2:**
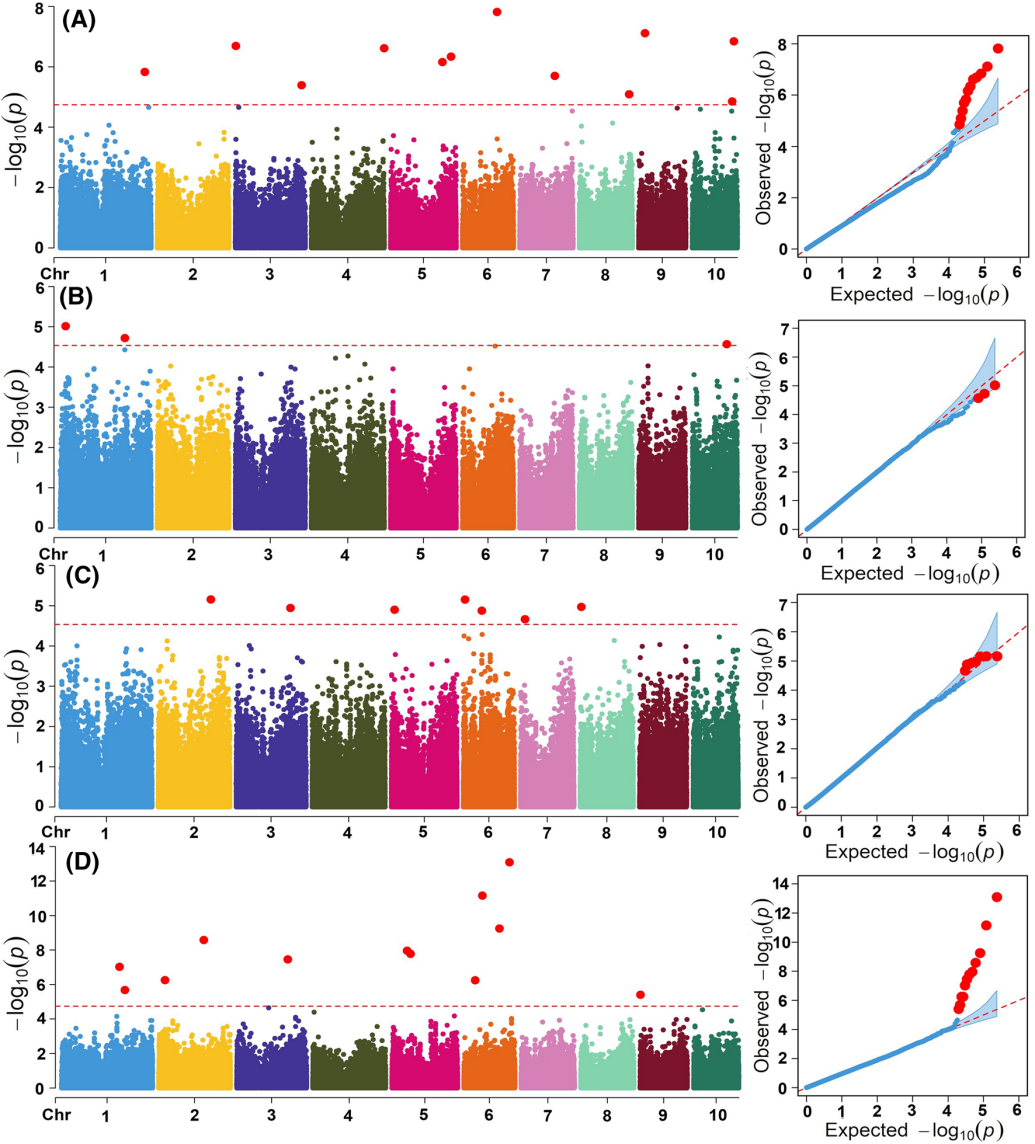
Manhattan and quantile–quantile plots generated using a mixed linear model for grain yield (**A**), Protein content (**B**), starch content (**C**) and Oil content (**D**) under optimum management. The significance level (*P* = 2 × 10^–5^ at *0.1 False Discovery Rate (FDR)*) is represented by the dashed horizontal line. The *X*-axis shows the position of SNPs along the 10 maize chromosomes, with various colours indicating distinct chromosomes. The *Y*-axis shows the − log10(*P* observed) in each analysis

**Fig. 3:**
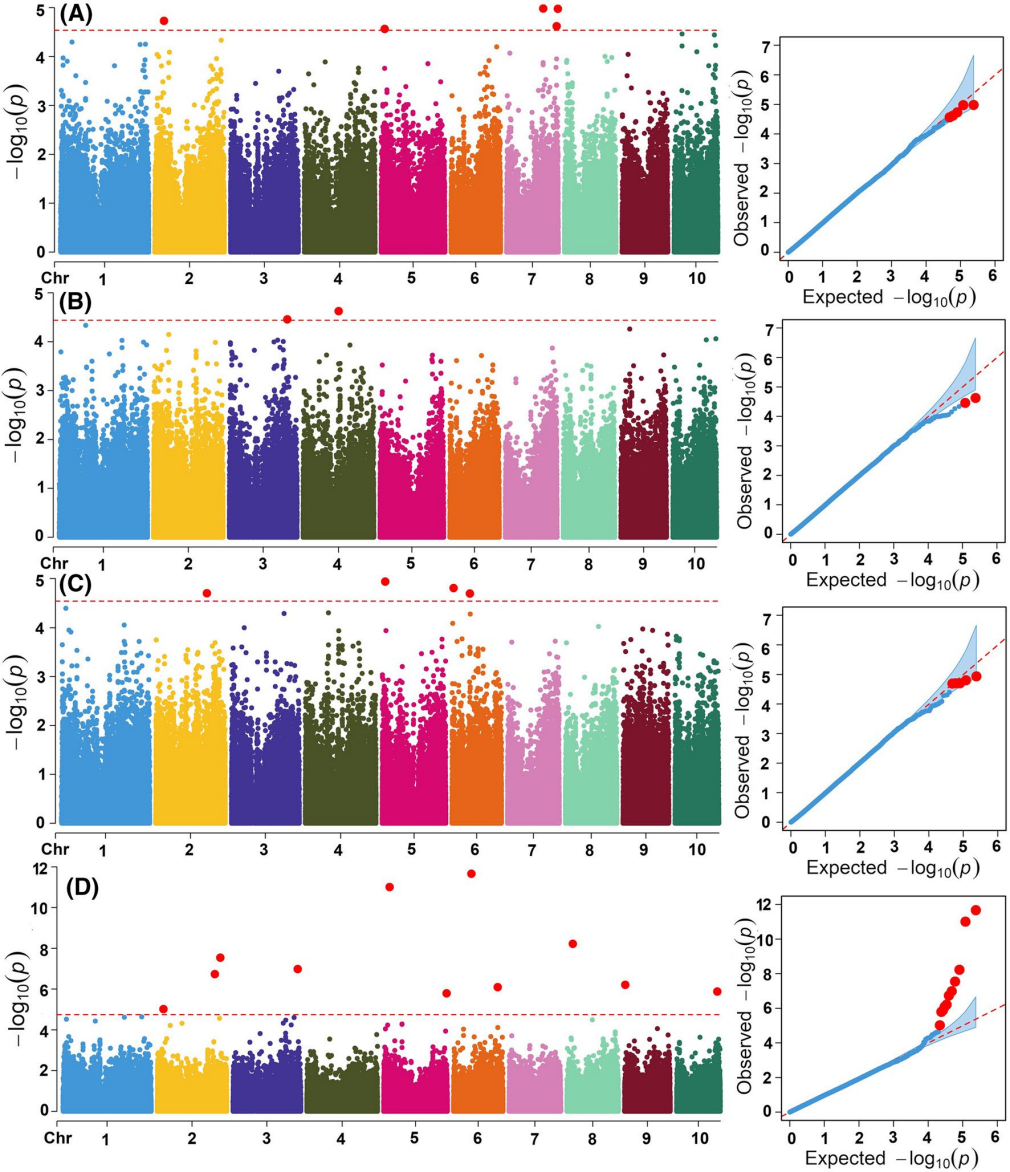
Manhattan and quantile–quantile plots generated using a mixed linear model for grain yield (**A**), protein content (**B**), starch content (**C**) and oil content (**D**) under low N stress management. The significance level (*P* = 2 × 10^–5^ at *0.1 False Discovery Rate (FDR)*) is represented by the dashed horizontal line. The *X*-axis shows the position of SNPs along the 10 maize chromosomes, with various colours indicating distinct chromosomes. The *Y*-axis shows the − log10(*P* observed) in each analysis

From GBS, 337,113 SNPs were produced for the 410 genotypes, in which 77.1% (259,798) remained after filtering using the > 5% MAF and 10% missing per marker criteria (Supplementary Figure S1). The kinship relations among the IMAS panel were determined using the filtered 259,798 SNP markers and depicted as a genetic cluster, indicating that the panel of genotypes are split into four potential genetically differentiated subgroups. The heatmap of the panel’s kinships was used to predict the magnitude of the existing relationships in the genotypes: this established that the genotypes were not closely related and that there is no strong population structure (Supplementary Figure S2). Further partition of the population structure of the IMAS panel using STRUCTURE 2.3.4 is presented in an earlier study by Kibe et al. (2020a) and Gowda et al. (2015). PCA was carried out using 259,798 high-quality SNPs (Supplementary Figure S3). The first principal component (PC1) accounted for roughly 4.5% of the overall variation, whereas the second principal component (PC2) explained 2.5% (Supplementary Figure S3). Calculation of genome-wide LD using 259,798 SNPs showed a significant decline in LD as genetic distance rose, with different rates of attenuation for each of the ten chromosomes (Supplementary Figure S4). Association analyses for grain yield and quality traits evaluated under optimum management were performed to evaluate the effects of different models on the control of false associations (Supplementary Figure S5). For all four traits, the observed *P* values from the GLM(G) and GLM (G + PCA) models showed the higher deviation from the expected *P* values is possibly due to either no associations or more false positives were detected. The *P* values from the MLM (PCA + K) and FarmCPU models were similar and close to the expected *P* values and are more effective in controlling the false associations (Supplementary Figure S5). With MLM model, between Kinship and some of the markers, the confounding effect is more severe and may results into overfitting of the model. On the other hand, FarmCPU model which uses both the fixed effect model and the random effect model iteratively, able to completely remove the confounding from kinship by using a fixed-effect model without a kinship derived either from all markers, or associated markers. This process overcomes the model overfitting problems of stepwise regression (Liu et al. 2016). Therefore, further in this study we used the results only FarmCPU model for both optimum and low N management conditions. Figures 2 and 3 depict the GWAS findings for protein, starch, and oil content, and yield across the two N managements as Manhattan and Q-Q plots of *p*-values evaluating the anticipated and observed − log10 *p*-values. Sixty-one SNPs were significantly (*P* = 2 × 10^–5^, *p* = 0.1 False Discovery Rate (FDR)) associated with the protein, starch, and oil content, and yield under optimal conditions and were spread across 10 chromosomes (Table 4). Under low N conditions, 42 SNPs are linked to the aforementioned traits.

**Table 4:** Chromosomal positions and SNPs significantly associated with grain yield, protein, starch, and oil content detected by SNP-based

SNP	Chr	Position	MLM-P value	MAF	Allele effect	Putative candidate genes	Predicted function of candidate gene
*Grain yield under optimum N*							
S1_279913054	1	279913054	1.46E-06	0.06	0.12	GRMZM2G300990	Protein serine/threonine kinase activity
S3_2218349	3	2218349	2.01E-07	0.09	0.10	GRMZM2G159307	ATP binding and magnesium chelatase activity
S3_220130640	3	220130640	4.03E-06	0.20	0.06	GRMZM2G100911	Protein serine/threonine kinase activity
S4_240579103	4	240579103	2.41E-07	0.23	0.07	GRMZM2G491366	Unknown
S5_171518940	5	171518940	6.87E-07	0.24	0.06	GRMZM2G043250	Zinc ion binding
S5_200110933	5	200110933	4.55E-07	0.33	− 0.06	GRMZM2G144898	Sexual reproduction
S6_114082980	6	114082980	1.51E-08	0.24	− 0.08	GRMZM2G165969	Serine-type endopeptidase activity
S7_115546635	7	115546635	1.98E-06	0.39	− 0.05	AC189790.2_FG003	Unknown
S8_164576912	8	164576912	8.04E-06	0.19	− 0.06	GRMZM2G121440	Shoot apex development
S9_20607559	9	20607559	7.62E-08	0.23	− 0.08	GRMZM2G391473	Protein serine/threonine kinase activity
S10_132510876	10	132510876	1.39E-05	0.39	− 0.05	AC194695.3_FG003	Unknown
S10_138021889	10	138021889	1.42E-07	0.07	0.11	GRMZM2G061287	Regulation of DNA-templated transcription
*Grain yield under low N*							
S2_30999187	2	30999187	1.85E-05	0.21	− 0.15	GRMZM2G052078	Shoot apex and pollen development
S5_11883140	5	11883140	2.72E-05	0.35	0.13	GRMZM2G090435	Homoiothermy
S7_124403566	7	124403566	1.04E-05	0.34	0.14	GRMZM2G007249	1-Aminocyclopropane-1-carboxylate oxidase activity
S7_170251419	7	170251419	2.39E-05	0.23	0.14	GRMZM2G024484	NADH dehydrogenase (ubiquinone) activity
S7_174752436	7	174752436	1.05E-05	0.08	− 0.24	GRMZM2G108166	DNA binding and helicase activity
*Protein content under optimum N*							
S1_17679954	1	17679954	9.57E-06	0.14	− 0.13	GRMZM2G410623	Shoot apex and pollen development
S1_214242607	1	214242607	1.90E-05	0.14	− 0.15	GRMZM2G127429	Aconitate hydratase activity
S10_114836465	10	114836465	2.69E-05	0.28	0.10	GRMZM2G049681	Shoot apex and pollen development
*Protein content under low N*							
S3_198394847	3	198394847	3.47E-05	0.17	0.13	GRMZM2G037102	Unknown
S4_120988951	4	120988951	2.36E-05	0.27	0.11	GRMZM2G032314	Shoot apex and pollen development
*Starch content under optimum N*							
S2_174345463	2	174345463	6.92E-06	0.21	− 0.16	GRMZM2G033694	Histone-lysine N-methyltransferase activity
S2_174345465	2	174345465	6.92E-06	0.21	− 0.16	GRMZM2G033694	Histone-lysine N-methyltransferase activity
S3_180044790	3	180044790	1.13E-05	0.15	− 0.17	GRMZM2G493214	Unknown
S5_10542862	5	10542862	1.25E-05	0.18	0.15	GRMZM2G114789	Nucleic acid-binding
S6_5158703	6	5158703	6.97E-06	0.30	− 0.15	GRMZM2G125138	Shoot apex and pollen development
S6_60978968	6	60978968	1.32E-05	0.09	− 0.22	GRMZM2G171810	Unknown
S7_14465153	7	14465153	2.14E-05	0.21	0.17	GRMZM2G104325	ATP biosynthesis process
S8_3430590	8	3430590	1.06E-05	0.13	− 0.18	GRMZM2G013461	Cysteine-type endopeptidase inhibitor activity
*Starch content under low N*							
S2_174345463	2	174345463	1.99E-05	0.21	− 0.17	GRMZM2G033694	Histone-lysine N-methyltransferase activity
S2_174345465	2	174345465	1.99E-05	0.21	− 0.17	GRMZM2G033694	Histone-lysine N-methyltransferase activity
S5_10542862	5	10542862	1.15E-05	0.18	0.17	GRMZM2G114789	Nucleic acid-binding
S6_5158703	6	5158703	1.56E-05	0.30	− 0.16	GRMZM2G125138	Shoot apex and pollen development
S6_60978968	6	60978968	2.02E-05	0.09	− 0.24	GRMZM2G171810	Unknown
*Oil content under optimum N*							
S1_191845162	1	191845162	9.38E-08	0.15	− 0.07	GRMZM2G026696	Unknown
S1_209355590	1	209355590	2.08E-06	0.41	0.04	GRMZM2G059138	Pollen and leaf development
S2_20538422	2	20538422	5.52E-07	0.11	0.07	GRMZM2G702954	Unknown
S2_148879075	2	148,879,075	2.63E-09	0.14	− 0.08	AC211891.4_FG001	Zinc ion binding
S3_169317292	3	169317292	3.50E-08	0.19	− 0.07	GRMZM2G034423	Unknown
S5_50087909	5	50087909	1.09E-08	0.22	0.06	GRMZM2G132464	Hydrolase activity, hydrolyzing O-glycosyl compounds
S5_61427444	5	61427444	1.65E-08	0.17	− 0.07	GRMZM2G417435	Shoot apex and pollen development
S6_36950637	6	36950637	5.58E-07	0.09	− 0.08	GRMZM2G571740	Unknown
S6_60978968	6	60978968	6.93E-12	0.09	0.11	GRMZM2G171810	Unknown
S6_117596170	6	117596170	5.58E-10	0.30	0.06	GRMZM2G505444	Unknown
S6_150886775	6	150886775	8.06E-14	0.11	0.12	GRMZM2G077789	DNA binding and pollen development
S9_1260192	9	1260192	3.91E-06	0.50	− 0.04	GRMZM2G500782	Unknown
*Oil content under low N*							
S1_269023923	1	269023923	2.31E-05	0.10	− 0.06	GRMZM2G155767	Phosphorelay sensor kinase activity
S2_20859604	2	20859604	9.48E-06	0.20	0.05	GRMZM2G014400	Nucleic acid-binding and shoot apex development
S2_196110550	2	196110550	1.82E-07	0.37	− 0.05	GRMZM2G113618	Monooxygenase and indoleacetaldoxime dehydratase activity
S2_214956407	2	214956407	2.83E-08	0.24	− 0.07	GRMZM2G153877	Shoot apex and pollen development
S3_221547612	3	221547612	1.03E-07	0.45	− 0.05	GRMZM2G028037	Protein serine/threonine kinase activity
S5_20201057	5	20201057	9.72E-12	0.43	0.06	GRMZM2G070523	DNA binding and shoot apex development
S5_214518378	5	214518378	1.60E-06	0.48	− 0.04	GRMZM2G008834	Protein binding
S6_60978968	6	60978968	2.15E-12	0.09	0.12	GRMZM2G171810	Unknown
S6_150841558	6	150841558	8.01E-07	0.11	0.07	GRMZM2G378586	Shoot apex and pollen development
S8_19834231	8	19834231	5.96E-09	0.15	0.07	AC218957.3_FG003	Unknown
S9_2472485	9	2472485	6.15E-07	0.30	− 0.05	GRMZM5G833563	DNA polymerase and nucleotidylexotransferase activity
S10_139315324	10	139315324	1.32E-06	0.19	0.05	GRMZM2G080516	DNA-binding transcription factor activity

GWAS in the IMAS association mapping panel under optimum and low N management conditions

*MAF Minor allele frequency; *MAE* minor allele effect; ^a^The exact physical position of the SNP can be inferred from marker’s name, for example, S1_82702920: chromosome 1; 82,702,920 bp (Ref Gen_v2 of B73)

Under optimal conditions, three SNPs linked with protein content were significant on chromosomes 1 (*S1_17679954* and *S1_214242607*) and 10 (*S10_114836465*). Under low N stress, however, two different SNPs *S3_198394847* and *S4_120988951* were associated with protein content. Starch content (low N) was associated with five SNPs, the most significant of which was *S5 10542862*. Eight SNPs on chromosomes 2 (*S2_174345463* and *S2_174345465*), 3 (*S3_180044790*), 5 (*S5_10542862*), 6 (*S6_5158703* and *S6_60978968*), 7 (*S8_3430590*), and 8 (*S7_14465153*) were linked with the starch content under optimum conditions. Under optimal conditions, twelve SNPs were significantly associated with oil content, with one-third of these loci located on chromosome 6. Twelve SNPs, with loci on all chromosomes except 4 and 7, were significantly linked with oil content under low N. For starch and oil content at low N conditions, only the significant SNP on chromosome 6 (*S6_60978968*) was co-detected. The proportion of detected SNPs for the other traits are presented in Table 4. The Q-Q plot for grain yield and oil content under optimum conditions and oil content under low N stress revealed that some observed *P*-values were more significant as the marker points migrated from the dotted red line towards the *y*-axis.

To elucidate the molecular and physiological mechanisms controlling grain quality traits under optimum and low N conditions, candidate genes were identified (Harper et al. 2016). On all chromosomes, a total of 51 candidate genes were discovered (Table 4). The lowest number of candidates (2) and the highest number (12) were related to protein content under low N and oil content under both optimum and low N, respectively. From these candidates, 80.39% (41 genes) were functionally annotated, whereas 19.61% (10 genes) were classified as unknown proteins. The study revealed four candidate genes with protein serine/threonine kinase activity that play a role in soil N response. Under optimum conditions, GRMZM2G159307 and GRMZM2G104325 were encoded as ATP binding proteins for grain yield and starch content, respectively. GRMZM2G10816 (yield), GRMZM2G070523 and GRMZM2G080516 (oil content) were associated with DNA biosynthesis under low N stress conditions. Under both optimal and low N circumstances, GRMZM2G033694 was annotated in the Histone-lysine N-methyltransferase family. Genes coding for shoot apex development were discovered to be associated with grain yield, protein, starch, and oil content under low N stress.

### QTLs associated with grain yield and quality traits

The four populations used in this study for linkage mapping were also used in our earlier study (Ertiro et al. 2020a) which includes detailed information about genetic maps. Table 5 shows the detected QTLs and their positions and genetic effects. In DH pop CML550/CML504, two QTLs each were detected for grain yield and starch content, three QTL for protein content and five QTL for oil content under low N stress. The PVE by these QTL was varied from 4.67 to 22.19% and together the total PVE was varied from 12.5% for grain yield to 47.9% for oil content. In DH pop CML550/CML511, one QTL each were detected for grain yield, starch content and oil content under low N stress. In DH pop CML505x LaPostaSeqC7-F64-2-6-2-2, five QTL were detected for grain yield with one QTL on chromosome 3 having a major effect with 12.17% of PVE. For protein content nine QTL were detected with all individually having minor effects except a QTL on chromosome 3 with 11.78% of variance explained. For starch content, three QTL each were detected under optimum and low N conditions with two major effects QTL on chromosome 8. For oil content three QTL were detected under optimum and six QTL were identified under low N conditions with one common QTL on chromosome 2 across management conditions. Four major effect QTL were identified for oil content on chromosomes 2, 4 and 5 which explained > 10% of the phenotypic variation (Table 5). In DH pop CML536xLapostaSeqiaF64, one QTL each were detected for protein and oil content and three QTL were detected for starch content, with one major effect QTL at chromosome 4 which contributes 20.3% of phenotypic variation for oil content.

**Table 5:** Number of QTL detected for grain yield, grain protein content, starch content and oil content under optimum (opt) and low-nitrogen (LN) stress across environments in four DH populations

Trait name	QTL name	Chr	Position (cM)	Left marker	Right marker	LOD	PVE (%)	TPVE (%)	Add	Fav allele
*DH pop1 (CML550* × *CML504)*										
Grain yield_LN	*qGY7_10*	7	629	S7_7419076	S7_10288357	3.31	6.60	12.51	0.22	CML550
	*qGY8_15*	8	578	S8_14728619	S8_15994213	3.54	6.85		0.22	CML550
Protein content LN	*qPC1_115*	1	525	S1_112639745	S1_162463938	6.60	11.04	30.51	0.13	CML550
	*qPC4_185*	4	115	S4_184892495	S4_185566354	3.38	5.39		0.09	CML550
	*qPC8_10*	8	128	S8_7675588	S8_12273955	4.71	8.30		0.11	CML550
Starch content LN	*qSC1_24*	1	47	S1_24583363	S1_24676752	3.44	6.94	19.24	− 0.13	CML504
	*qSC4_174*	4	95	S4_173800701	S4_174954893	4.56	9.06		− 0.15	CML504
Oil content LN	*qOC1_268*	1	313	S1_268152564	S1_268431295	3.28	3.49	47.92	− 0.04	CML504
	*qOC3_03*	3	294	S3_3559699	S3_3912927	7.98	8.84		− 0.07	CML504
	*qOC6_60*	6	35	S6_56452467	S6_63537451	4.30	4.67		− 0.05	CML504
	*qOC6_133*	6	103	S6_132237821	S6_133515728	17.69	22.19		− 0.11	CML504
	*qOC8_165*	8	503	S8_164378522	S8_166561727	5.97	6.95		− 0.06	CML504
*DH pop2 (CML550* × *CML511)*										
Grain yield_LN	*qGY5_15*	5	610	S5_4278140	S5_202395053	2.75	8.79	8.62	− 0.74	CML511
Starch content LN	*qSC1_180*	1	288	S1_175655223	S1_187977553	2.75	11.52	11.45	0.22	CML550
Oil content LN	*qOC3_60*	3	206	S3_58480638	S3_156799630	2.65	10.47	9.78	− 0.09	CML511
*DH pop3 (CML505 *×* LapostaSequiaF64)*										
Grain yield Opt	*qGY3_187*	3	207	S3_186485761	S3_189293786	3.40	9.47	8.86	− 0.037	LPSF64
Grain yield LN	*qGY3_196*	3	175	S3_195927184	S3_202609108	9.91	12.17	26.28	− 0.093	LPSF64
	*qGY3_207*	3	954	S3_206952639	S3_208331031	3.92	4.41		0.063	CML505
	*qGY4_160*	4	28	S4_159978522	S4_160277631	3.99	4.59		− 0.063	LPSF64
	*qGY4_155*	4	524	S4_154382211	S4_155910715	6.79	8.06		0.076	CML505
Protein content_Opt	*qPC4_140*	4	162	S4_125492338	S4_141413020	3.04	8.46	14.15	− 0.002	LPSF64
	*qPC4_180*	4	577	S4_179648189	S4_180236732	10.68	4.23		− 0.003	LPSF64
	*qPC5_70*	5	291	S5_69971990	S5_73044866	4.16	8.46		− 0.002	LPSF64
	*qPC6_157*	6	57	S6_156035854	S6_157412461	3.95	4.23		− 0.002	LPSF64
	*qPC6_40*	6	652	S6_36947497	S6_45922975	3.04	4.23		− 0.002	LPSF64
	*qPC7_126*	7	166	S7_125835683	S7_127144774	4.59	8.46		0.003	CML505
	*qPC10_142*	10	102	S10_141963062	S10_142398096	3.92	8.46		0.002	CML505
Protein content_LN	*qPC3_187*	3	208	S3_180516308	S3_189293786	5.27	11.78	24.15	0.076	CML505
	*qPC4_146*	4	193	S4_145319458	S4_147746777	3.63	7.73		− 0.062	LPSF64
Starch content_Opt	*qSC2_14*	2	47	S2_13290784	S2_18626409	3.93	8.63	23.99	0.056	CML505
	*qSC4_205*	4	677	S4_204860243	S4_206663242	3.42	3.70		0.049	CML505
	*qSC5_14*	5	380	S5_13609325	S5_14471659	3.77	8.63		0.051	CML505
Starch content_LN	*qSC8_123*	8	294	S8_122410298	S8_123512730	11.35	7.00	19.95	− 0.313	LPSF64
	*qSC8_124*	8	298	S8_123512730	S8_124357554	18.85	12.86		0.427	CML505
	*qSC9_24*	9	224	S9_23280315	S9_30187968	3.32	1.73		0.176	CML505
Oil content_Opt	*qOC2_186*	2	480	S2_185692862	S2_186464021	4.89	10.53	35.22	0.030	CML505
	*qOC4_70*	4	506	S4_68323590	S4_75504829	6.01	12.56		− 0.032	LPSF64
	*qOC5_182*	5	158	S5_182450446	S5_181213050	4.35	8.94		− 0.028	LPSF64
Oil content_LN	*qOC2_186*	2	482	S2_185692862	S2_186464021	6.12	7.87	54.86	0.042	CML505
	*qOC3_223*	3	87	S3_224567900	S3_222406989	3.35	4.14		− 0.031	LPSF64
	*qOC4_60*	4	188	S4_57947900	S4_75164940	3.42	4.32		− 0.031	LPSF64
	*qOC5_183*	5	148	S5_183857938	S5_182051683	11.99	17.31		− 0.063	LPSF64
	*qOC7_08*	7	470	S7_8930324	S7_7716737	7.89	10.71		0.052	CML505
	*qOC9_80*	9	263	S9_79412157	S9_83242552	7.44	9.87		− 0.048	LPSF64
*DH pop4_CML536* × *LapostaSequiaF64*										
Protein content _LN	*qPC5_67*	5	197	S5_68309122	S5_66022006	3.12	16.04	15.90	0.051	CML536
Starch content_LN	*qPC2_08*	2	255	S2_8288623	S2_10208	3.09	6.16	37.02	0.105	CML536
	*qSC4_32*	4	245	S4_36777242	S4_31674554	9.80	20.36		0.200	CML536
	*qSC9_135*	9	127	S9_135814387	S9_134561093	5.20	9.65		0.138	CML536
Oil content_LN	*qOC5_21*	5	229	S5_21876475	S5_20198056	3.40	8.25	8.19	− 0.049	LPSF64

*Chr Chromosome, *LOD* Logarithm of Odds; *add* additive effect; *PVE* phenotypic variance explained; *fav allele* parental line contributing the favourable allele for trait, QTL name composed by the trait code followed by the chromosome number in which the QTL was mapped and a physical position of the QTL

Comparison of the QTLs across association mapping panel and DH populations revealed several QTLs overlapped for same traits for across optimum and low N conditions, as well as for multiple traits (Supplementary Figure S5, S6). In Chromosome 1, two regions, between 209 to 214 Mb and 268 to 280 Mb had QTL for more than one trait. In chromosome 2, bin 2.03 harboured QTL for both GY and oil content, whereas bin 2.06 has QTL for both Starch content and oil content under both optimum and low N conditions. At chromosome 3, bin 3.06 had QTL for grain yield, protein content, oil content and starch content (Supplementary Figure S5). Further QTL clustering for more than one trait was observed on chromosome 4, bin 3.06 and 3.07, on chromosome 5, at bin 5.02 and 5.05, on chromosome 6, at bin 6.02 and 6.06 and on chromosome 10 at bin 10.06. Significant SNP, *S1_269023923* associated with oil content detected through GWAS was co-located with QTL (*qOC_01_269*) detected in DH pop1. Another SNP *S5_11883140* detected for GY in GWAS panel was co-located with QTL *qGY_05_15* detected on DH pop2 (Tables 4 and 5).

**Fig. 4:**
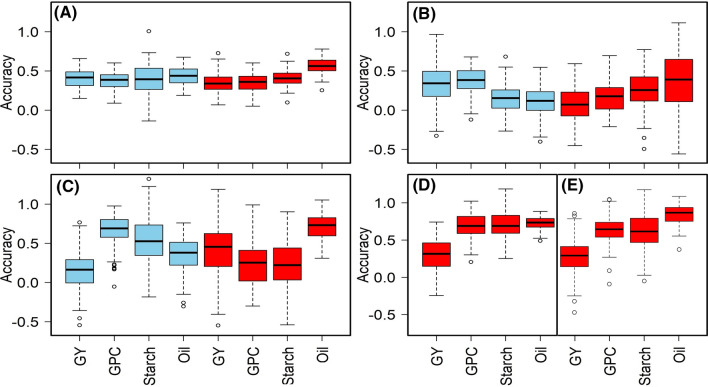
Genome-wide prediction accuracies for grain yield and quality traits in IMAS panel (**A**), DH pop2 = CML550/CML511 (**B**), DH pop3 = CML505/LaPostaSeqC7-F64-2-6-2-2 (**C**), DH pop1 = CML550/CML504 (**D**) and DH pop4 = CML536/LaPostaSeqC7-F64-2-6-2-2 (**E**). Blue and red colour box plots indicate traits were evaluated under optimum and low N stress conditions, respectively (color figure online)

The RR-BLUP model (Endelman 2011) was used to estimate the performance of maize genotypes for grain quality traits for each population (Fig. 4 and Supplementary Table S2). Under low nitrogen conditions, average prediction accuracies across the studied genotypes were higher for oil content (0.78) and lower for grain yield (0.08). In IMAS panel we observed the prediction accuracy of 0.41, 0.38. 0.39 and 0.44 under optimum and 0.35, 0.35, 0.41 and 0.56 under low N conditions, respectively. Interestingly, in DH pop CML550/CML504 outperformed other DH populations in terms of genomic prediction accuracy. Under low N, CML550/CML504 had the best prediction accuracy for protein (0.66), oil (0.73), and starch (0.7) content. The prediction accuracy for protein content was highest in DH pop CML505/LaPostaSeqC7-F64-2-6-2-2 under optimum (*r* = 0.69) and for DH pop CML550/CML504 under low N stress (*r* = 0.66). For starch content under low N, prediction correlation was highest for CML550/CML504 (*r* = 0.70) followed by the CML536x LaPostaSeqC7-F64-2-6-2-2 (*r* = 0.56), IMAS panel (*r* = 0.41), CML550xCML511 (*r* = 0.26) and CML505x LaPostaSeqC7-F64-2-6-2-2 (*r* = 0.23). CML536x LaPostaSeqC7-F64-2-6-2-2 had the highest prediction correlation for oil content under low N (*r* = 0.78), followed by CML550/CML504 (*r* = 0.73) and CML505x LaPostaSeqC7-F64-2-6-2-2 (*r* = 0.71). CML550xCML511 had the lowest prediction for grain yield (*r* = 0.08), protein (*r* = 0.17) starch (*r* = 0.16), and oil content (*r* = 0.11).

## Discussion

Significant levels of malnutrition (Christian and Dake 2021) and food insecurity (Giller 2020) continue to be experienced by maize-dependent smallholder farming populations in SSA that cultivate in nitrogen-depleted soils. Unravelling the genetic architecture of grain yield and quality traits through GWAS and GS is critical for the development of superior genotypes conferring high expression of grain quality traits both under optimum and low N stress. This study aimed to understand the underlying genetics of low N stress on grain quality traits by combining GWAS with the IMAS panel, QTL detection, and GS in four bi-parental populations. Grain quality traits, notably protein, starch, and oil content, are critical for reducing the incidence of undernutrition in SSA. Understanding the performance of grain quality traits and associated genetic markers under low N stress can aid in the development of maize lines with high protein, starch, and oil content.

### Phenotypic evaluation under optimum and low N stress

Phenotypic analyses showed that protein, starch, and oil content were significantly decreased under low N stress compared to optimal conditions across all tested genotypes. This is consistent with the findings of Liu et al. (2008), who found that in lower N conditions, protein and oil content are considerably reduced. However, the same research reported the opposite for starch content increased under lower levels of N stress. According to Jahangirlou et al. (2021) and Simić et al. (2020), high N conditions are associated with higher protein content and yield. On the other hand, low N substantially decreases protein concentration and all zein fractions apart from β-zeins, according to research conducted in Zimbabwe by Shawa et al. (2021). The impact of soil nutrient management on oil content was significant in the study of Ray et al. (2019). That study reported that using N as a component in NPK blends increased the quantity of saturated fatty acids while decreasing the percentage of unsaturated fatty acids in grain maize oil. Kaplan et al. (2017) suggested that N fertilizer application in combination with adequate irrigation has a favourable effect on the oil content. However, in SSA due to the impoverished economic situation of smallholder farmers, N fertilization is not currently an accessible solution to combat endemic undernutrition. Therefore, maize lines showing high protein, starch and oil content under low N stress should be considered for incorporation into maize breeding programs targeting the SSA region.

In low N stress environments, genetic variability is crucial for the effective selection of enhanced grain quality traits in maize. Ertiro et al. (2020a) asserted that, due to the intrinsic unpredictability of various traits of interest, phenotypic data for trials conducted under low N conditions typically show poor heritability. However, under both optimum and low N environments, our study estimated wide genetic variances and moderate to high broad sense heritabilities. Estimates of heritability ranging from moderate to high imply that the traits have the potential to be enhanced by recurrent selection (Gowda et al. 2021). The influence of G, E, and G x E interactions on oil content was significant under low N conditions across all genotypes examined. The genotypic effects on protein content were significant in some of the genotypes tested (CML505/LaPostaSeqC7-F64-2-6-2-2 and CML536/LaPostaSeqC7-F64-2-6-2-2) under low N conditions. The G × E effects of CML550/CML511 on protein and starch content were significant. The detected significant genotypic variation for the assessed traits in this study indicated the possibility of selecting for improved protein, starch, and oil content under low N stress. Among the three grain quality traits investigated, starch content had the lowest *H*^*2*^ estimate, whereas oil content had the highest *H*^*2*^ estimate under low N conditions. Oil content’s high broad-sense heritability suggests that its narrow-sense heritability may be even greater, implying that significant genetic gain for this trait is attainable.

Grain yield had a negative genotypic correlation with protein content in all populations, regardless of N level. This supports Arisede et al. (2020)’s findings that increased grain yield was associated with decreased grain protein content in both susceptible and tolerant maize hybrids when residual soil N was low, despite the fact that tolerant hybrids showed a substantially smaller loss in grain protein content. It is well known that choosing between yield and quality is hard in breeding. The observed relationship between grain yield and quality traits in this research under both optimal and low N conditions imply that selecting for grain yield alone will not increase protein, starch, or oil content. On the other hand, protein content had a significant negative correlation with starch content in all populations, which is consistent with previous results (Liu et al. 2008; Zheng et al. 2021). Thus, to increase grain quality, particularly under low N stress, there is a need to select for both grain yield and grain quality. Obviously, this would be very expensive, and a negative relationship makes breeders balance in the selection of these traits hence the need to investigate the potential of molecular breeding in the improvement of these traits.

### Association mapping and candidate genes

Association studies targeting protein, oil, and starch content in maize have been conducted utilizing a range of genotypes and marker sets (Alves et al. 2019; Cook et al. 2012; Zheng et al. 2021). The use of GWAS in maize genetics has been highly effective in discovering causal genes for grain quality traits (Zheng et al. 2021). In particular, GWAS is an effective technique for mapping loci linked with complex plant traits in genetically heterogeneous populations (Deng et al. 2021). The power of detection of GWAS is dependent on the LD between the markers and QTL. In outcrossing plant species such as maize, LD declines at a short distance and rapidly (Dinesh et al. 2016). In this study, the LD declined rapidly across physical distance (Kibe et al. 2020a), showing that the IMAS panel has significant genetic diversity and was, therefore, suitable for GWAS.

Candidate genes and SNPs discovered by GWAS for maize grain nutrient content can provide critical information for maize breeding efforts focusing on developing high-quality varieties (Zheng et al. 2021). In this study, GWAS identified 42 SNPs linked to the grain quality traits studied under low N conditions. However, there were no overlapping SNPs for grain quality traits under low N. Under low N stress, two SNPs on chromosomes 3 (*S3_198394847*) and 4 (*S4_120988951*) were discovered to be linked to protein content. Moreover, 12 SNPs with loci on all chromosomes except 4 and 7 were also shown to be substantially associated with oil content under low N stress. The genetic regions identified in this work through GWAS will be increasingly relevant in future breeding approaches for accurate selection of high grain quality and to increase tolerance of maize lines to low N stress.

Comparison of SNPs identified in this study under low N and optimum conditions revealed no overlapping of SNPs for grain yield and protein content possibly we were not able to detect the common variants responsible for these traits in different management conditions. Nonetheless, for starch content, all the SNPs detected under low N were also detected under optimum conditions. Whereas for the oil content SNP *S6_60978968* on chromosome 6 was consistently detected in both the conditions and also found common SNPs in bin 2.01, 5.03, 6.05 and 9.01 on chromosome 2, 5, 6 and 9, respectively (Table 4). Further comparison of the detected SNPs with the previous studies revealed some overlapping with earlier reported QTL (Wang et al. 2016; Zheng et al. 2021). For instance, SNP *S1_214242607* associated with grain protein content under optimum was closely located with marker detected through GWAS (Zheng et al. 2021) and co-located with QTL detected in two populations (Wang et al. 2016). SNP *S1_191845162* detected for oil content was co-located within the QTL (bnlg2086-umc1122 interval) reported by Zhang et al. (2008) and Wang et al. (2010, umc1395-umc2237 interval). Marker *1_190758142* detected through GWAS for oil content by Zheng et al. (2021) was also located within the same QTL region pointing to the importance of the region for improving oil content in maize. Another SNP *S2_148879075* detected for oil content was located within the QTL region (bnlg108-phi092 interval) reported by Zhang et al. (2008). SNP *S1_17679954* for protein content was co-located within the QTL detected for oil content on chromosome 1 (umc1685-umc1044 interval) in F_3_ population (Wang et al. 2010). Nevertheless, some SNPs did not coincide with earlier reports in terms of their physical location. This possibly due to several reasons like these SNPs might be specific to the population in this study, the variation for quality traits in these populations is different, and different methods used to estimate quality traits in different studies also contribute to variation. However, new specific SNPs detected in this study need further validation, nevertheless, these results can serve as a reference for future studies.

We identified 51 candidate genes potentially underlying the molecular and physiological processes governing grain quality traits under optimum and low N environments. The identification of candidate genes based on associated SNPs can aid with the identification of genes important in grain quality performance under optimal and low N environments. Under low N stress, genes coding for shoot apex growth were revealed to be linked with grain yield, protein, starch, and oil content. Peng et al. (2010) asserted that shoot growth, rather than root size, is a good indicator of N sufficiency in maize. The research also identified four candidate genes with protein serine/threonine kinase activity that play a role in soil N response. Protein kinases are well-known regulators of the response of plants to abiotic stresses (Diédhiou et al. 2008; Kulik et al. 2011; Mao et al. 2010). GRMZM2G159307 and GRMZM2G104325 encode ATP binding proteins for grain yield and starch content, respectively. ATP binding proteins are essential for cellular motility, membrane transport and the control of different metabolic activities (Chauhan et al. 2009). ATP-binding has also been reported in several studies to influence the maintenance of homeostasis in plants under both abiotic and biotic stresses (Dahuja et al. 2021; Franz et al. 2011; Jarzyniak and Jasiński 2014). GRMZM2G033694 was assigned to the Histone-lysine N-methyltransferase family at both optimum and low N conditions. It is important to note, however, that these candidate genes should be further validated before being used in breeding schemes. Further functional research on the candidate genes discovered in this study is necessary to validate their possible utility in high grain quality breeding under low N conditions.

### Linkage mapping on grain yield and quality traits

Linkage mapping in four populations found multiple QTLs for the studied grain quality traits. Zheng et al. (2021) alluded that, numerous grain nutritional quality QTLs in maize have been identified by genetic dissection of nutrient quality over the last two decades using traditional QTL mapping. Despite the discovery of QTLs and genes that confer superior maize grain quality in some studies, further sources of genetic variation are likely to exist among currently unexplored populations. QTL analyses in four DH populations revealed 8, 13, 12 and 15 potential QTLs associated with grain yield, protein, starch, and oil content, respectively. One QTL on chromosome 3 (*qGY3_187*) for grain yield is overlapped with major effect QTL (*qPC3_187*) for protein content and located between 180 and 189 Mb, which might be an interesting region to improve both protein and grain yield by considering their negative relationship. Zhang et al. (2015) also identified a consistent QTL (umc1644-phi102228 interval) in the same genomic region for protein content. Another QTL *qPC1_115* in CML550/CML504 which explained 11% of the phenotypic variance was consistent with earlier reported QTL (phi001-umc1988 interval) by Zhang et al. (2015) and *qPC10_142* detected on chromosome 10 was consistent with QTL (SYN37373—PZE110095199 interval) reported by Wang et al. (2016) in recombinant inbred line population. There was one major effects QTL (> 10% phenotypic variance explained) for grain yield (*qGY3_196*), three QTL each for protein content (*qPC1_115*, *qPC3_187*, *qPC5_67*) and starch content (*qSC1_180*, *qPC4_32*, *qPC8_124*) and six QTLs for oil content (*qOC2_186*, *qOC3_60*, *qOC4_70*, *qOC5_183*, *qOC6_133*and *qOC7_08*) were detected in four biparental populations. A major QTL (*qSC1_180*) identified in DH pop CML550xCML511, explaining about 11.5% of total phenotypic variance and located between 175 and 188 Mb, was consistent with a QTL (SYN367-PZE101031077 interval) observed in a RIL population by Wang et al. (2016). Similarly, another QTL for starch content (*qSC8_124*) located between 123 and 124 Mb also coincided with earlier reported QTL on chromosome 8 (PZE108069534-SYN19928 interval; Wang et al. (2016)). Similarly, major QTL for oil content *qOC2_186* was also overlapped with earlier detected QTL*,* indicating several consistent regions for quality traits across genetic back grounds which supports their stable nature and is amenable for MAS-based improvement. Overall, several QTLs were consistent with the previous studies indicating their reliability to be used in applied breeding.

Comparison of QTLs detected in both GWAS, and linkage mapping revealed clusters on several chromosomes either for same trait under both optimum and low N management as well as for both grain yield and quality traits (Supplementary Figure S5 and S6). Clustering of QTL for grain yield for both under optimum and low N conditions were detected on chromosome 3 between 186 to 200 Mb in DH pop3 and another QTL between 207 to 220 Mb in DH pop3 and GWAS panel. Several SNPs detected in GWAS were co-located within the QTLs detected in DH populations, for instance like for GY on chromosome 6 at 12 Mb and oil content at chromosome 6 at 60 Mb, these results help in further reducing the confidence interval of these QTLs. Focusing on increasing the favourable alleles associated with QTL in this region helps to improve the QTL for both low N and optimum management conditions. Similarly on chromosome 4, region between 140 to 180 Mb harbours QTL for grain yield, protein content and starch content and on chromosome 5 in region between 10 to 21 Mb harbours QTL for grain yield, starch content and oil content. These regions are of most important for simultaneous improvement of both grain yield and quality traits for both the management conditions. Nevertheless, further reducing the confidence interval of these regions helps to get more strongly associated markers for these QTLs which enhance the success rate to improve the multiple traits for both optimum and low N management.

### Genomic predictions on grain yield and quality traits

GS in tropical maize for various traits of interest revealed moderate to high prediction accuracies in several studies (Azmach et al. 2018; Beyene et al. 2019; Crossa et al. 2014; Gowda et al. 2021). The relative merits of GS over phenotypic selection influence its widespread application in breeding programs ( Beyene et al. 2019, 2021; Kibe et al. 2020a). Moderate to high accuracies observed in this study for the bi-parental populations and IMAS panel offer promise in breeding for quality traits in tropical maize. Under N-starved soils, average prediction accuracies (Fig. 4) were higher for oil content (0.78) and lower for grain yield (0.08) which ascribed to their differences in their genetic architecture as oil content is relatively less complex in nature. DH population CML550/CML504 exhibited the highest prediction accuracy for protein (0.69), oil (0.73), and starch (0.70) content under low N stress. GS prediction accuracy has a direct influence on the degree of trait variation and heritability in each population (Kibe et al. 2020a). This is confirmed by this study, especially for oil content which had the highest genetic prediction accuracy is agrees with high magnitude of genotypic variation and *H*^*2*^ estimates. Prediction accuracy for quality traits in the IMAS panel was in agreement with various studies on moderately complex traits like resistance for grey leaf spot (Kibe et al. 2020a), common rust (Kibe et al. 2020b), *Striga* (Gowda et al. 2021), maize lethal necrosis and maize chlorotic mottle virus (Sitonik et al. 2019). In the IMAS panel, the observed moderate prediction accuracy can be attributed to its genetic structure and high LD between adjacent markers, which could also be credited to its moderate heritability. Overall, this study indicates that utilizing a common training population to predict grain quality trait performance under low N stress in many linked but separate populations can be beneficial. In addition, the results also suggest that for complex traits like GY, selective marker-based approaches are less effective to improve their performance, however MAS can help to improve for quality traits irrespective of the management conditions.

Compared to grain yield, quality traits are less complex, however, improving them under low N stress conditions through traditional breeding is laborious. Further improvement of these traits through MAS or MABC, there is need of more validation experiments for each trait to confirm the identified genomic regions for their consistency under diverse genetic background and environment, and fine map these regions to have stable markers which is resource intensive. On the other hand, GS is not needed any prior information on trait specific markers but is efficient in predicting lines performance on desired traits. In addition, the major QTL information available through QTL mapping, and GWAS can be incorporated into GS model as fixed effects which further enhance the prediction accuracy for these traits and helps to improve the efficiency in developing high yielding and high protein and oil content genotypes for optimum as well as low N stress conditions. Therefore, integration of GS in breeding program is beneficial to improve multiple traits.

## Conclusions

To investigate the genetic basis of protein, starch, and oil content performance under low N stress, we employed a single panel consisting of 410 tropical maize lines for GWAS and genomic prediction. QTL mapping was also used to investigate the underlying genetic architecture in four bi-parental populations to better understand the grain quality traits. The genotypic correlations of the grain quality traits investigated indicated that these populations can be used to select better-performing lines under low N stress. GWAS identified 42 SNPs associated with grain quality traits. In addition, several QTLs for the examined grain quality traits were identified by linkage mapping across populations. The genomic regions identified can be used for selection efforts to enhance grain quality trait performance in low-nitrogen soils. Furthermore, the findings showed that including GS in maize breeding can successfully support phenotypic selection to improve grain quality trait performance under low N stress. Future work should, therefore, focus on validating the identified QTLs to enhance the efficacy of maize breeding in SSA.

## Supplementary Information

Below is the link to the electronic supplementary material.

Supplementary file1 (DOCX 1006 kb)

Supplementary file2 (XLSX 213 kb)

## Data Availability

All datasets generated for this study are included in the article/Supplementary material. The GBS marker data are available at https://data.cimmyt.org/file.xhtml?persistentId=hdl:11529/10548467/1.
