# Supplementary material for: Genome-wide association studies of grain yield and quality traits under optimum and low-nitrogen stress in tropical maize (Zea mays L.)
Source: Theor Appl Genet. 2022 Sep 21;135(12):4351–70. doi: 10.1007/s00122-022-04224-7 (PMC9734216; doi:10.1007/s00122-022-04224-7)
Supplement: Supplementary file 1 — Supplementary file1 (DOCX 1006 kb) [file 122_2022_4224_MOESM1_ESM.docx]

**Supplementary materials**


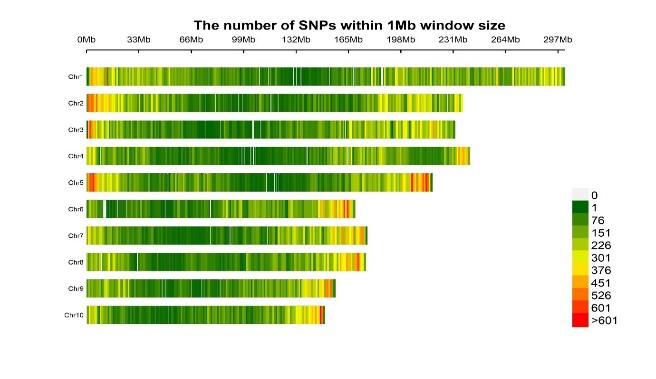


**Supplementary Figure S1.** Density plot showing the number SNPs within 1 Mb window size from the studied 259,798 markers. The number of SNPs is displayed on a scale from green to red.


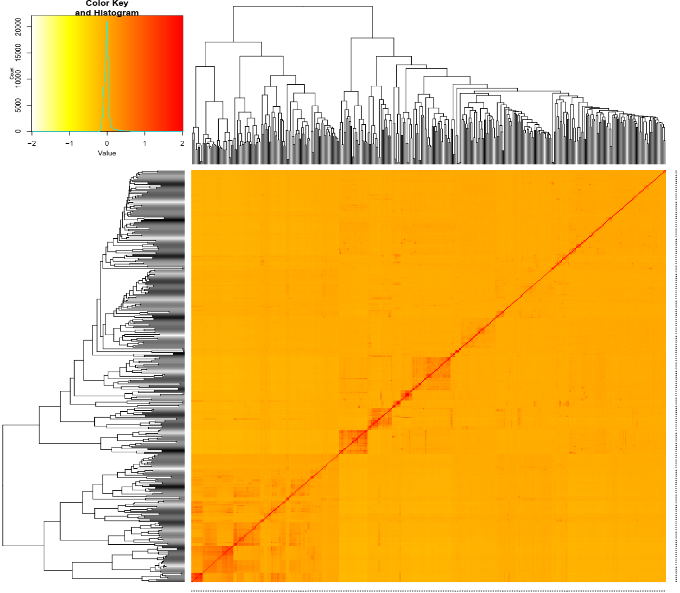


**Supplementary Figure S2.** **Kinship Analysis of the IMAS panel**. The heat map shows the pairwise kinship matrix based on 259,798 filtered SNPs.

**A B**


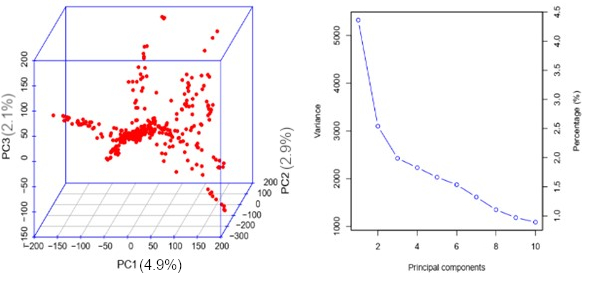


**Supplementary Figure S3.** (A) Population structure representation based on the first three principal components (PC). (B) Scree plot of the estimated eigen values of each PC. The PCA plots of the IMAS panel were computed using 259,798 SNPs of the IMAS panel.


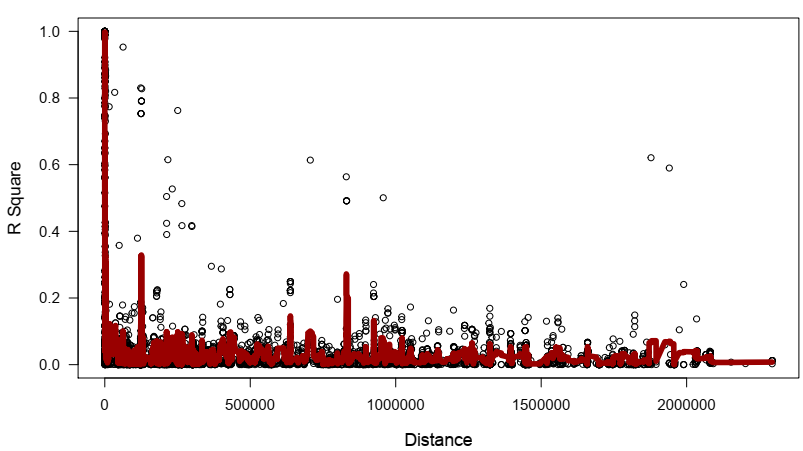


**Supplementary Figure S4** LD decay plot for the whole genome-based on 259,798 SNPs. The x-axis values represent the physical distance (kb), while the y-axis values represent the squared correlation coefficient (r^2^).


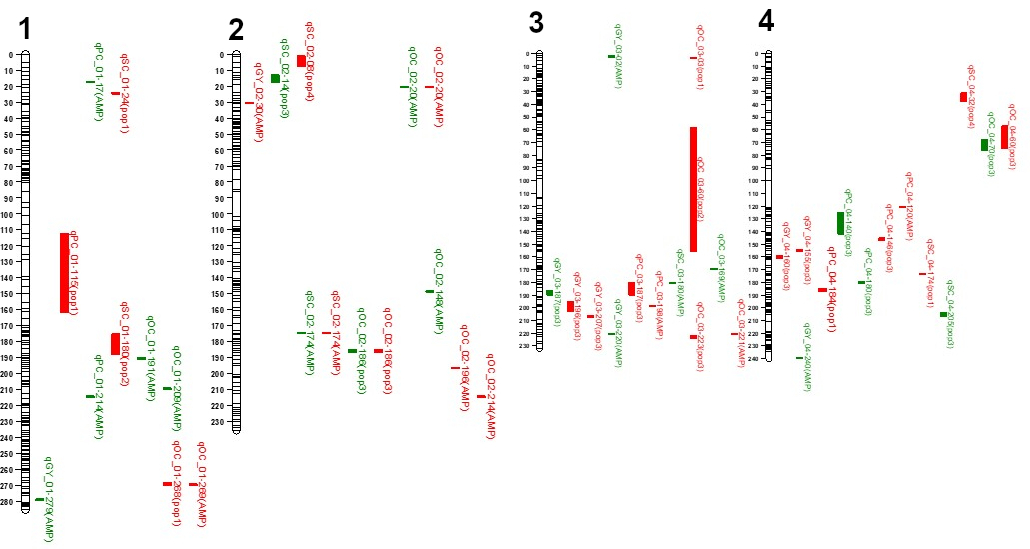


**Supplementary Figure S5.** QTL on chromosome 1, 2, 3 and 4 for grain yield (GY), protein content (PC), starch content (SC) and oil content (OC) in the consensus linkage map of four DH populations. Green and red color represents QTL detected under optimum and low N conditions, respectively.


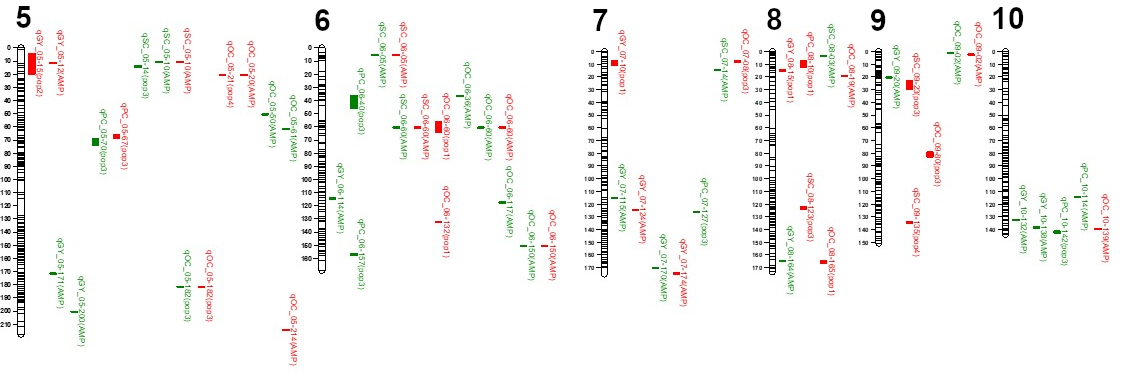


**Supplementary Figure S6.** QTL on chromosome 5, 6, 7, 8, 9 and 10 for grain yield (GY), protein content (PC), starch content (SC) and oil content (OC) in the consensus linkage map of four DH populations. Green and red color represents QTL detected under optimum and low N conditions, respectively.


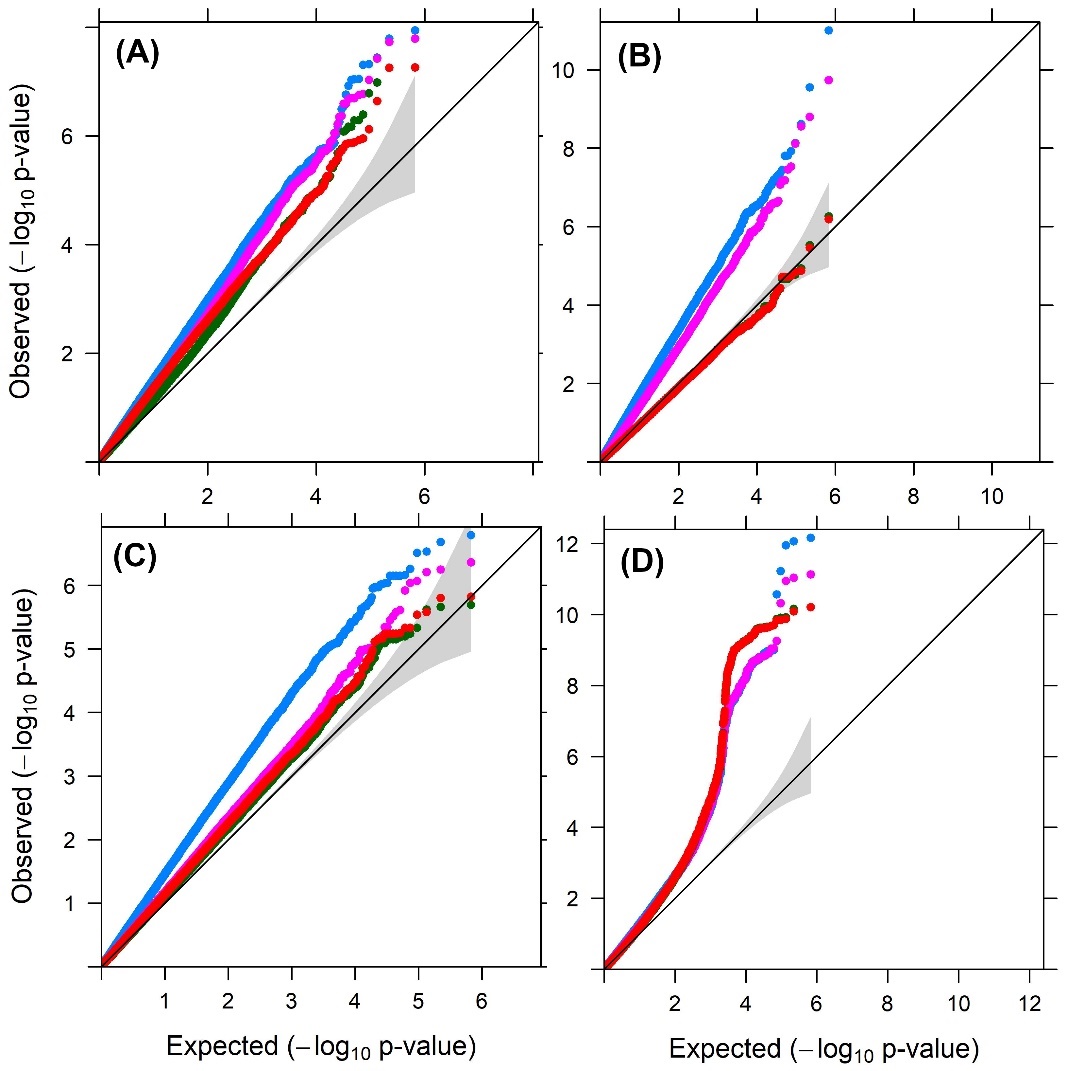


**Supplementary Figure S7.** Q-Q plots of the estimated -log10(P) from association panel for four traits. A, B, C and D represent Q-Q plots for grain yield, protein content, starch content and oil content based on the BLUP values across multiple environments under optimum management conditions, respectively. The black line bisecting the plot represents the expected P values with no associations present. The blue line represents observed P values using the simplest model GLM(G) where the association between a phenotype and markers are directly detected. The pink line represents observed P values using the GLM (PCA+G) model. The green line represents observed P values using the MLM (PCA+K+G) model. The red line represents observed P values using the FarmCPU model. G = genotype (fixed), PCA = three principal components (fixed), K = kinship matrix (random).

**Supplementary Table S1.** SNP distribution, proportion of missing markers and heterozygous markers across ten chromosomes.

| Chromosome | Number of SNPs | | Proportion Missing (%) | | | Proportion Heterozygosity | | | Mean |
| --- | --- | --- | --- | --- | --- | --- | --- | --- | --- |
|  | **Raw Data** | **Filtered** | **Min** | **Max** | **Mean** | **Min** | **Max** | **Mean** |  |
| 1 | 52,977 | 40,910 | 0 | 0.99 | 0.003 | 0 | 0.31 | 0.095 | 0.22 |
| 2 | 41,550 | 32,083 | 0 | 0.99 | 0.003 | 0 | 0.31 | 0.096 | 0.22 |
| 3 | 38,701 | 29,694 | 0 | 0.99 | 0.003 | 0 | 0.31 | 0.093 | 0.22 |
| 4 | 31,020 | 23,849 | 0 | 0.99 | 0.003 | 0 | 0.30 | 0.092 | 0.22 |
| 5 | 39,463 | 30,411 | 0 | 0.99 | 0.003 | 0 | 0.31 | 0.095 | 0.22 |
| 6 | 27,130 | 21,053 | 0 | 0.99 | 0.003 | 0 | 0.31 | 0.093 | 0.22 |
| 7 | 28,593 | 21,991 | 0 | 0.99 | 0.003 | 0 | 0.30 | 0.091 | 0.22 |
| 8 | 28,816 | 22,192 | 0 | 0.99 | 0.003 | 0 | 0.31 | 0.094 | 0.23 |
| 9 | 25,290 | 19,500 | 0 | 0.99 | 0.003 | 0 | 0.28 | 0.094 | 0.22 |
| 10 | 23,573 | 18,115 | 0 | 0.99 | 0.003 | 0 | 0.29 | 0.092 | 0.22 |
| TOTAL | **337,113** | **259,798** |  | | | | | | |

**Min, minimum; max, maximum. The recorded parameters are presented for SNPs after filtering using the 5% MAF and 10% missing criterion*

**Supplementary Table S2.** Genome-wide prediction accuracies for grain yield and quality traits

|  | IMAS Panel | DH pop1 | DH pop2 | DH pop3 | DH pop4 |
| --- | --- | --- | --- | --- | --- |
| **Optimum** | | | | | |
| Grain yield | 0.41 | - | 0.33 | 0.14 | - |
| Protein content | 0.38 | - | 0.38 | 0.66 | - |
| Starch content | 0.39 | - | 0.16 | 0.53 | - |
| Oil content | 0.44 | - | 0.11 | 0.35 | - |
| **Low N** | | | | | |
| Grain yield | 0.35 | 0.31 | 0.08 | 0.41 | 0.28 |
| Protein content | 0.35 | 0.69 | 0.17 | 0.69 | 0.60 |
| Starch content | 0.41 | 0.70 | 0.26 | 0.23 | 0.56 |
| Oil content | 0.56 | 0.73 | 0.36 | 0.71 | 0.78 |

DH pop1= CML550/CML504; DH pop2= CML550/CML511; DH pop3= CML505/LaPostaSeqC7-F64-2-6-2-2; and DH pop4= CML536/LaPostaSeqC7-F64-2-6-2-2.

**Supplementary Table S3.** Percentage change in grain yield and quality traits between optimum and Low N stress conditions

| Genotype | Grain yield (%) | Protein content (%) | Starch content (%) | Oil content (%) |
| --- | --- | --- | --- | --- |
| IMAS | -58.81 | -23.41 | 2.57 | -4.42 |
| DH pop1 | -51.36 | -23.13 | 2.61 | 2.24 |
| DH pop2 | -47.58 | -27.19 | 2.25 | -6.87 |
| DH pop3 | -46.89 | -8.43 | 1.02 | 3.84 |
| DH pop4 | -55.32 | -27.71 | 3.49 | 0.09 |

*A negative value indicates that the percentage change is decreasing, whereas a positive number indicates the opposite. DH pop1= CML550/CML504; DH pop2= CML550/CML511; DH pop3= CML505/LaPostaSeqC7-F64-2-6-2-2; and DH pop4= CML536/LaPostaSeqC7-F64-2-6-2-2
